# *OSBPL2* encodes a protein of inner and outer hair cell stereocilia and is mutated in autosomal dominant hearing loss (*DFNA67*)

**DOI:** 10.1186/s13023-015-0238-5

**Published:** 2015-02-10

**Authors:** Michaela Thoenes, Ulrike Zimmermann, Inga Ebermann, Martin Ptok, Morag A Lewis, Holger Thiele, Susanne Morlot, Markus M Hess, Andreas Gal, Tobias Eisenberger, Carsten Bergmann, Gudrun Nürnberg, Peter Nürnberg, Karen P Steel, Marlies Knipper, Hanno Jörn Bolz

**Affiliations:** Institute of Human Genetics, University Hospital of Cologne, Cologne, Germany; Molecular Physiology of Hearing, Hearing Research Centre Tübingen (THRC), Department of Otolaryngology, University of Tübingen, Tübingen, Germany; Department of Phoniatrics and Pediatric Audiology, Hannover Medical School, Hannover, Germany; Wolfson Centre for Age-Related Diseases, King’s College London, London, UK; Cologne Center for Genomics (CCG) and Center for Molecular Medicine Cologne (CMMC), University of Cologne, Cologne, Germany; Institute for Human Genetics, Hannover Medical School, Hannover, Germany; Department of Voice, Speech and Hearing Disorders, University Medical Center Hamburg-Eppendorf, Hamburg, Germany; Department of Human Genetics, University Medical Center Hamburg-Eppendorf, Hamburg, Germany; Center for Human Genetics, Bioscientia, Ingelheim, Germany; Renal Division, Department of Medicine, University Medical Center Freiburg, Freiburg, Germany; Cologne Excellence Cluster on Cellular Stress Responses in Aging-Associated Diseases (CECAD), University of Cologne, Cologne, Germany

**Keywords:** *OSBPL2*, *DFNA67*, Autosomal dominant hearing loss

## Abstract

**Background:**

Early-onset hearing loss is mostly of genetic origin. The complexity of the hearing process is reflected by its extensive genetic heterogeneity, with probably many causative genes remaining to be identified. Here, we aimed at identifying the genetic basis for autosomal dominant non-syndromic hearing loss (ADNSHL) in a large German family.

**Methods:**

A panel of 66 known deafness genes was analyzed for mutations by next-generation sequencing (NGS) in the index patient. We then conducted genome-wide linkage analysis, and whole-exome sequencing was carried out with samples of two patients. Expression of Osbpl2 in the mouse cochlea was determined by immunohistochemistry. Because *Osbpl2* has been proposed as a target of *miR-96*, we investigated homozygous *Mir96* mutant mice for its upregulation.

**Results:**

Onset of hearing loss in the investigated ADNSHL family is in childhood, initially affecting the high frequencies and progressing to profound deafness in adulthood. However, there is considerable intrafamilial variability. We mapped a novel ADNSHL locus, *DFNA67*, to chromosome 20q13.2-q13.33, and subsequently identified a co-segregating heterozygous frameshift mutation, c.141_142delTG (p.Arg50Alafs*103), in *OSBPL2*, encoding a protein known to interact with the *DFNA1* protein, DIAPH1. In mice, Osbpl2 was prominently expressed in stereocilia of cochlear outer and inner hair cells. We found no significant *Osbpl2* upregulation at the mRNA level in homozygous *Mir96* mutant mice.

**Conclusion:**

The function of *OSBPL2* in the hearing process remains to be determined. Our study and the recent description of another frameshift mutation in a Chinese ADNSHL family identify *OSBPL2* as a novel gene for progressive deafness.

**Electronic supplementary material:**

The online version of this article (doi:10.1186/s13023-015-0238-5) contains supplementary material, which is available to authorized users.

## Background

Hearing impairment is the most common sensory disorder, affecting approximately 1/500 newborns. In developed countries, most cases are of genetic origin, and there is extensive allelic and non-allelic heterogeneity. In 70% of hearing-impaired neonates, the sensory deficit is non-syndromic (non-syndromic hearing loss, NSHL). Approximately 20% of patients have autosomal dominantly-inherited forms (ADNSHL) and typically display postlingual progressive hearing impairment. Sixty-five ADNSHL loci have been officially designated, and 30 causative genes have been reported [1-3]. Because of the extensive genetic heterogeneity of hearing impairment, the identification of the causative mutation in single patients has been the exception until recently. With the advent of next-generation sequencing (NGS), deafness genes have become accessible to comprehensive genetic analysis and routine genetic testing by targeted NGS of “gene panels” [4].

## Methods

### Patients

**Figure 1:**
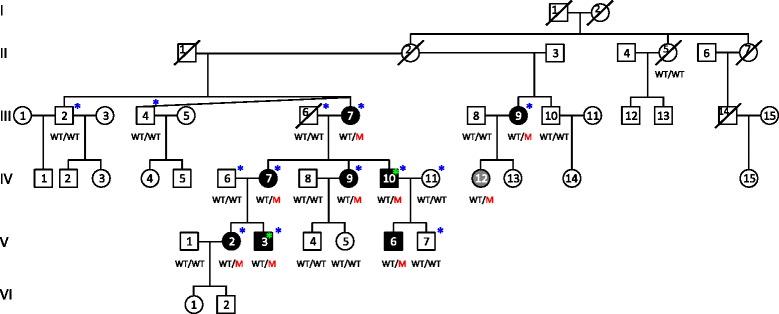
**Pedigree and genotypes of the** ***DFNA67*** **family.** M, *OSBPL2* mutation; WT, wildtype. Blue stars, individuals whose samples were subjected to genome-wide SNP genotyping for linkage analysis. Green stars, individuals whose samples were subjected to WES.

Samples of the German family reported herein (Figure 1) were obtained with written informed consent. Clinical investigations were conducted according to the Declaration of Helsinki, and the study was approved by the institutional review board of the Ethics Committee of the University Hospital of Cologne. The affected subjects underwent detailed audiological evaluations (e.g., pure tone audiometric air conduction, bone conduction, speech reception threshold, otoacoustic emissions, and impedance audiometry, phoneme discrimination), except IV:12 who reports intermittent hearing impairment (related to stress). Following the recommendations of the EU HEAR project [5], hearing loss was classified as mild (20 – 40 dB), moderate (41 – 70 dB), severe (71 – 95 dB), or profound (>95 dB). This classification, however, was difficult in some cases, because at least two patients (V:2, V:3) had near normal hearing in the 250 – 1000 Hz region, but a loss of about 70 – 90 dB in the higher frequencies.

### *GJB2* analysis and targeted NGS of a deafness gene panel

The *GJB2* gene was directly sequenced in the index patient, IV:10. His sample was then subjected to NGS for 66 genes (1,259 coding exons) that have been associated with NSHL and selected forms of SHL on a MiSeq system (Illumina) as described previously [6]. In brief, sheared DNA was ligated to bar-coded adaptors for multiplexing. Exons were targeted by an in-solution customized sequence capture library (NimbleGen). Amplified enriched DNA was directly subjected to NGS (MiSeq). Reads were mapped against the hg19 human reference genome using BWA [7] and processed with SAMtools [8], Picard (http://broadinstitute.github.io/picard/) and GATK [9]. Variants were filtered against dbNSFP v2.0 [10], dbSNP v137, the Human Gene Mutation Database (HGMD® Professional 2013.2) [11] and our in-house database. The cutoff for the maximum minor allele frequency (MAF) was set to 1% [12]. Nonsense, frameshift and canonical splice site variants were regarded likely pathogenic. SNVs were assessed using SIFT [13], Mutation Taster [14], PolyPhen-2 [15], AlignGVGD [16,17], Pmut [18], NNSPLICE v0.9 [19] and NetGene2 [20,21]. SeqPilot SeqNext module (v4.0.1, JSI medical systems) was used for visualization and final assessment of SNVs.

### Linkage analysis and locus designation

DNA samples from seven affected and six unaffected members of the family (Figure 1) were genotyped using the Affymetrix GeneChip Human Mapping 10 K SNP array Xba142. The gender was verified by counting heterozygous single nucleotide polymorphisms (SNPs) on the X chromosome. Relationship errors were evaluated with the help of the program Graphical Representation of Relationships [22]. Linkage analysis was performed assuming autosomal dominant inheritance, full penetrance, and a disease gene frequency of 0.0001. Multipoint LOD scores were calculated using the program ALLEGRO [23]. Haplotypes were reconstructed with ALLEGRO and presented graphically with HaploPainter [24]. All data handling was performed using the graphical user interface ALOHOMORA [25].

Following the identification of the causative mutation in *OSBPL2*, *DFNA67* was assigned as a novel locus designation for ADNSHL by the Human Gene Nomenclature Committee, HGNC.

### Whole-exome sequencing

Samples of two affected family members, IV:10 and V:3, were subjected to whole-exome sequencing (WES). DNA (1 μg) was fragmented using sonication technology (Covaris). Fragments were enriched using the SeqCap EZ Human Exome Library v2.0 kit (Roche NimbleGen) and subsequently sequenced on an Illumina HiSeq 2000 sequencing instrument using a paired-end 2 × 100-bp protocol. This generated 6.1 and 8.2 Gb of mapped sequences with a mean coverage of 80-fold and 101-fold, a 30-fold coverage of 89% and 89% of the target sequences and a 10-fold coverage of 96% and 95% of the target sequences. For data analysis, the Varbank pipeline (v.2.3) and filter interface was used (https://varbank.ccg.uni-koeln.de). Primary data were filtered according to signal purity by Illumina Real-Time Analysis (RTA) software (v1.8). Subsequently, the reads were mapped to the human genome reference build hg19 using the bwa-aln alignment algorithm. GATK v1.6 [9] was used to mark duplicated reads, perform local realignment around short insertions and deletions, recalibrate the base quality scores, and call SNPs and short indels. Scripts developed in-house at the CCG were applied to variants predicted to result in protein changes, variants affecting donor and acceptor splice sites, and those overlapping with known variants. Acceptor and donor splice site mutations were analyzed with a maximum-entropy model and filtered for their putative effect on splicing. In particular, we focused on the linkage regions (chr12:49,329,157-52,752,362 and chr20:52,882,032-61,366,354; hg19) and filtered for high-quality (>15-fold coverage; quality > 25; VQSR > -2), and for rare (MAF <0.005) and heterozygous variants. Several genome-wide databases (dbSNP Build 135, 1000 Genomes Project database build 20110521 [26] and the public Exome Variant Server, NHLBI, Seattle, build ESP6500 [27]) were checked for the presence of the identified variants. To exclude pipeline-related artifacts (MAF <0.005), we also filtered against an in-house database containing variants from 511 exomes of individuals with epilepsy. Finally, we filtered for variants present in both patients. Verification of sequence variants and segregation analyses were carried out by Sanger sequencing. Sequence data for *OSBPL2* (MIM 606731) were compared to the reference sequence NM_144498.2.

### RT-PCR from cochlear tissue

mRNA from mouse postnatal day 19 (P19) and rat P17 cochleae was isolated as described earlier [28]. A 298 bp fragment of *Osbpl2* was amplified using the following primer sequences: 5′-CCAACTCTGCTCAGATGTACAAC-3′ (forward) and 5′-GCTGTACGCCGGCCATTACTTTGA-3′ (reverse). PCR was performed with PuReTaq^TM^ Ready-To-Go^TM^ PCR beads (GE Healthcare). The amplification conditions consisted of an initial denaturation phase of 94°C for 4 min, 35 cycles of 30 s denaturation (94°C), 30 s annealing (58°C), 30 s extension (72°C), and a final extension phase of 5 min at 72°C. The PCR products were analyzed on ethidium bromide agarose gels. Fragments were extracted using QIAquick Gel Extraction Kit (Qiagen), cloned and sequenced.

### Tissue preparation and immunohistochemistry

Cochleae from adult mice (aged between P20 and six months) were used to prepare cryosections for immunofluorescence microscopy. For immunohistochemistry on cryosections, the temporal bone of mature mice was dissected on ice and immediately fixed using Zamboni’s fixative [29] containing picric acid by infusion through the round and oval window and incubated for 15 min on ice, followed by rinsing with phosphate-buffered saline (PBS) and decalcification in rapid bone decalcifier (Eurobio, Fisher-Scientific). After injection of 25% sucrose in PBS, pH 7.4, cochleae were embedded in O.C.T. compound (Miles Laboratories, Elkhart, IN). Tissues were then cryosectioned at 10 μm thickness, mounted on SuperFrost*/plus microscope slides, dried for 1 h and stored at -20°C before use. Cryosections were thawed and permeabilized with 0.1% Triton X-100 (Sigma Aldrich) for 3 min at room temperature, blocked with 1% BSA in PBS, and incubated with primary antibody in 0.5% BSA in PBS overnight at +4°C. For double labeling studies, specimens were simultaneously incubated with both antibodies for identical time periods. As primary antibodies we used: anti-ORP-2 (OSBPL2) antibody (goat, Santa Cruz Biotechnology, Inc., sc-66570, dilution 1:50), anti-prestin antibody (rabbit [30], dilution 1:5000), and anti-otoferlin antibody (rabbit [31], dilution 1:10000). Primary antibodies were detected with Cy3- (Jackson Immunoresearch Laboratories) and Alexa488- (Invitrogen, Life Technologies GmbH) conjugated secondary antibodies. Sections embedded with Vectashield mounting medium containing nuclear marker DAPI (Vector Laboratories). Sections were viewed using an Olympus BX61 microscope equipped with motorized z-axis and epifluorescence illumination. To increase display resolution, cochlear slices were imaged over a distance of several micrometers (30 × 0,27 μm, ~8 μm) in an image stack along the z-axis (z-stack), followed by 3-dimensional deconvolution using cellSens Dimension module with the advanced maximum likelihood estimation algorithm (ADVMLE; OSIS). Images were acquired using a CCD camera and analyzed with cellSens software (Olympus Soft Imaging Solutions, OSIS). Images were processed with Photoshop.

### Comparison of *Osbpl2* mRNA levels between wildtype and homozygous *Mir96* mutant *diminuendo* (*Mir96*^*Dmdo*^) mice

Organ of Corti dissection, RNA extraction and cDNA creation were carried out as described previously [32]. Primers were purchased from Applied Biosystems (*Hprt1*: Mm01318747_g1, *Jag1*: Mm01270190_m1, *Osbpl2*: Mm01210488_m1). *Hprt1* was used as an internal control, and *Jag1*, which is expressed in supporting cells [32-34], was used to control for the quantity of sensory tissue present. Pairs were only used when *Jag1* levels did not differ significantly between wildtype and homozygote littermates (p > 0.05). Three animals were genotyped and at least three technical replicates were performed on each pair. The reaction was run on a CFX Connect machine using Bio-Rad SsoFast and SsoAdvanced Master mixes (Bio-Rad Laboratories, cat. nos. 1725232, 1725281).

## Results

### Clinical characterization of *DFNA67* patients

**Figure 2:**
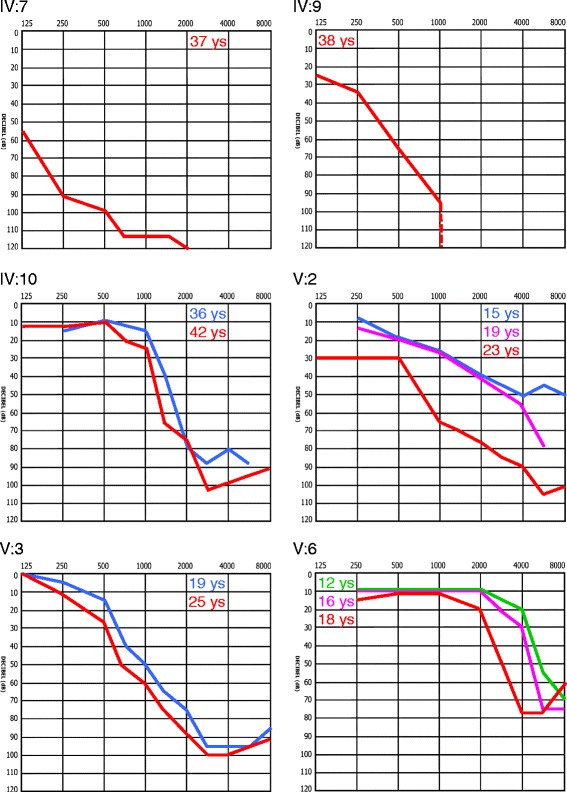
**Exemplary audiograms of patients from the** ***DFNA67*** **family.** Hearing thresholds are shown for the more severely affected side.

In most affected family members, bilateral sensorineural non-syndromic hearing loss was first noted in the early second decade of life and affected initially the high frequencies. However, the age of onset varied between 10 (patients IV:7, V:6) and 30 years of age (III:9). Real onset may have been earlier in many cases: Retrospectively, the parents of patient V:3 assume that hearing was already impaired around the age of 2 years. Progression of hearing loss was also widely variable. It was mild in younger individuals but severe to profound at later stages (Figure 2) and required cochlear implantation between 27 and 50 years of age in five family members. On the mild end of the spectrum, patient IV:10 noted onset of hearing loss at 22 years of age and started using hearing aids at 34 years. No audiological data were available for IV:12, a 39-years old carrier of the *OSBPL2* mutation, who does not use hearing aids but claims worse hearing in stressful situations. III:7 and V:2 reported progression of hearing loss in the course of pregnancy and birth of children (see Table 1 for a summary of clinical data). Vestibular symptoms were not reported.

**Table 1:** **Summary of clinical data**

**Individual**	**Age (ys.)**	**Hearing loss**	**Age of onset (ys.)**	**Course**	***OSBPL2***
II:1	36 (†)	no			no sample
II:2	69 (†)	not noted by any of the five children, but nasal pronunciation	?	?	no sample
II:5	93 (†)	no			wildtype
II:7	78 (†)	no			no sample
III:2	76	no			wildtype
III:4	71	unilateral (untreated otitis media)			wildtype
III:6	74 (†)	no			wildtype
III:7	71	yes	12	worse after birth of children, stress; CI around 50 ys.	p.Arg50Alafs*103
III:8	67	no			wildtype
III:9	61	yes	30	CI at 50 ys.	p.Arg50Alafs*103
III:10	64	only temporary, episodes of acute hearing loss			wildtype
IV:6	58	no			wildtype
IV:7	50	yes	10	CI at 36 ys.	p.Arg50Alafs*103
IV:8	50	no			wildtype
IV:9	49	yes	12	CI at 39 ys.	p.Arg50Alafs*103
IV:10	45	yes	22	hearing aids at 34 ys.	p.Arg50Alafs*103
IV:11	44	no			wildtype
IV:12	39	no data from investigations; hearing worse under stress	?	?	p.Arg50Alafs*103
IV:13	35	no; two healthy daughters			no sample
V:2	28	yes	15	worsening after birth of children; CI at 27 ys.	p.Arg50Alafs*103
V:3	26	yes	11	hearing aids at 12 ys.	p.Arg50Alafs*103
V:4	15	no			wildtype
V:5	16	no			wildtype
V:6	20	yes	10	hearing aids at 15 ys.	p.Arg50Alafs*103
V:7	17	no			wildtype
VI:1	3	no (normal OAEs at 3 ys.)			wildtype
VI:2	4 months	no			

### Mapping of chromosomal candidate loci

**Figure 3:**
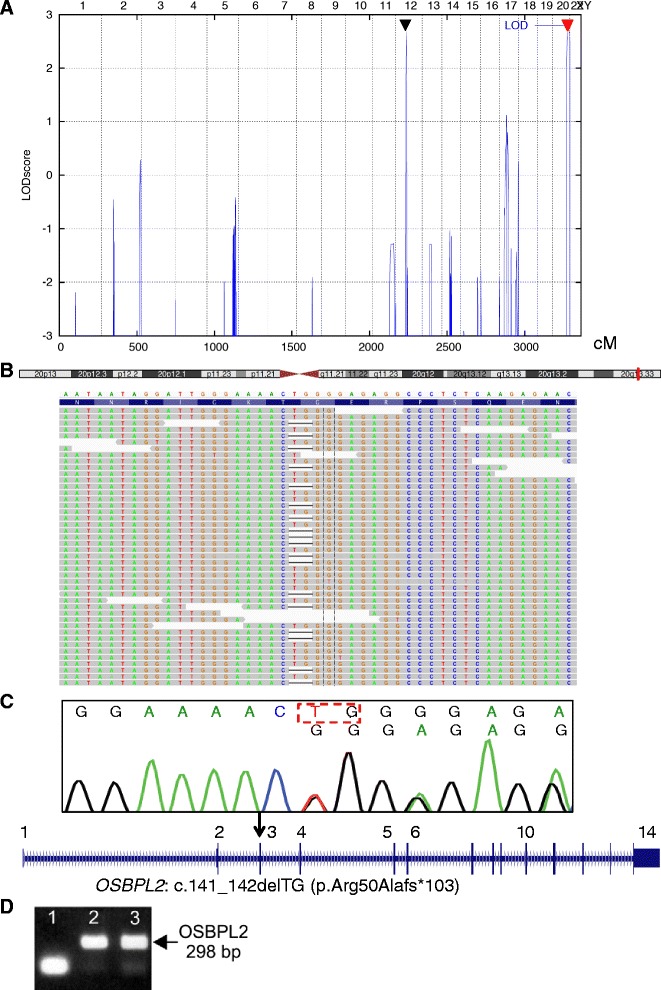
**Genetics of the German** ***DFNA67*** **family. A** Graphical view of the LOD score calculation of genome-wide SNP mapping. A 3.4 Mb region on chromosome 12 and an 8.4 Mb region on chromosome 20 showed potential linkage with the phenotype. **B** Ideogram of chromosome 20 with the position of *OSBPL2* indicated (red bar). Schematic representation of the mapped sequencing reads (forward strand) visualized with the *Integrative Genomics Viewer* (IGV) for patient IV:10. The c.141_142delTG (p.Arg50Alafs*103) mutation in *OSBPL2* was present in half of the reads covering this region of the gene. **C** Electropherogram of a heterozygous carrier of the *OSBPL2* mutation in exon 3 (deleted nucleotides are boxed). The localization of the mutation is indicated in a scheme of the *OSBPL2* gene. **D** RT-PCR demonstrates *Osbpl2* expression at the transcriptional level in mouse (lane 2) and rat (lane3) cochlea. Lane 1, no cDNA as negative control.

Using ALLEGRO we identifed two genomic regions with a maximum LOD score of 2.7. The obtained LOD score was the maximum possible LOD score in this family. We found linkage to a 3.4 Mb interval between SNPs rs1316607 and rs725029 on chromosome 12 (49,329,157 – 52,752,362) and to a 8.4 Mb interval between rs2065042 and rs720607 on chromosome 20 (52,882,032 – 61,366,354) (Figure 3 A).

### Targeted NGS, WES and segregation analysis of the candidate variants identified

No mutation was identified in *GJB2* and in subsequent targeted NGS of 66 known deafness genes. After stringent filtering of WES data, only two heterozygous variants remained that localized to the chromosome 12 candidate region: The c.1516C>G (p.Arg506Gly) variant in *SLC11A2* has been annotated in dbSNP (rs199589052), but no MAF is available. Biallelic mutations in *SLC11A2* cause autosomal recessive hypochromic microcytic anemia with iron overload but there is no mention of any hearing impairment [35]. It is therefore unlikely that heterozygous *SLC11A2* mutations cause ADNSHL. A missense variant, c.53G>A (p.Arg18His), was identified in *CELA1* (chymotrypsin-like elastase family, member 1). Because of its expression in skin tissue, *CELA1* had been considered a candidate for skin disease. However, a common frameshift polymorphism questioned essentiality of this gene [36]. Moreover, the p.Arg18His variant has an MAF of 0.28% which is not compatible with a mutation causing a rare autosomal dominant disorder.

There were two heterozygous missense variants in genes contained in the candidate region on chromosome 20: The variant c.1202G>C (p.Arg401Pro) in *GTPBP5* affects an evolutionarily non-conserved residue and has been annotated as a polymorphism, rs200118420, with an MAF of 0.04%. A variant in *DIDO1* (c.1738A>C; p.Thr580Pro), also affects a non-conserved residue. Mutant *Dido* -/- and +/- mice appeared grossly normal. With time, some heterozygous mice showed abnormalities in spleen, bone marrow, and peripheral blood, overlapping with symptoms of myeloid dysplasia or myeloid proliferation [37]. Taken together, none of these three missense variants appeared to be a promising candidate for the ADNSHL-causing mutation.

A nonsense mutation, c.287C>G (p.Ser96*), was found in both patients in *SLC17A9*, a gene directly adjacent (61,583,999 – 61,599,949) to the telomeric boundary of the chromosome 20 locus. According to the Exome Variant Server, the *SLC17A9* nonsense variant has an MAF of 0.06%, and compatible with the gene’s localization just outside the candidate locus, it was carried by three healthy individuals of the family (II:5, III:10, IV:13). Moreover, p.Ser96*_SLC17A9_ was also present in heterozygous state in three samples of our in-house database: A patient with epilepsy, a patient with amyotrophic lateral sclerosis and the healthy mother of that patient. None of these three individuals had hearing loss.

We identified only one truncating variant in a gene contained in a mapped candidate region: Both patients carried the frameshift mutation c.141_142delTG (p.Arg50Alafs*103) in *OSBPL2* (Figure 3B,C), a gene from the chromosome 20 region. This variant has not been annotated in any of the above databases, no allele frequency is available, and it co-segregated perfectly with hearing loss in the family.

### Expression of *Osbpl2* in the murine cochlea

**Figure 4:**
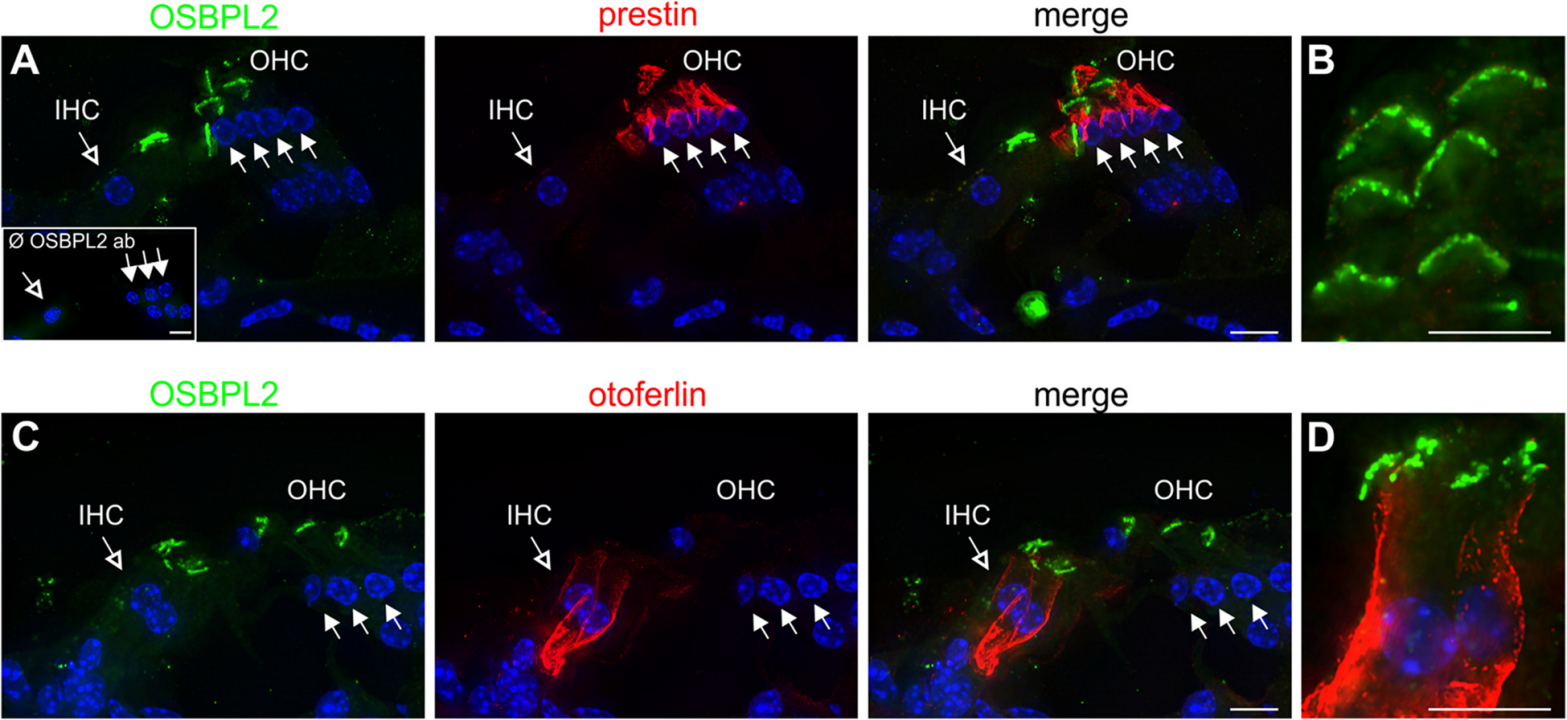
**OSBPL2 expression in stereocilia of mouse cochlear inner and outer hair cells. A**, **B** OSBPL2 (green) is expressed in stereocilia of cochlear outer hair cells (OHC) as demonstrated by co-immunostaining with anti-prestin antibody (red) of mature (P20) mice. Upon omission of the primary antibody no immunostaining can be seen, which demonstrates the specificity of the OSBPL2 antibody (inset). **C**, **D** OSBPL2 (green) is also expressed in stereocilia of cochlear inner hair cells (IHC) as demonstrated by co-immunostaining with anti-otoferlin antibody (red). Nuclei are stained with DAPI (blue). Scale bars, 10 μm.

We analyzed the expression of *Osbpl2* at the transcriptional level of post-hearing animals using RT-PCR with mRNA from whole mouse (P19) and rat (P17) cochlea. The amplification product of *Osbpl2* with the appropriate size (298 bp) was found in both mouse (Figure 3D, lane 2) and rat cochlea (Figure 3D; lane3). Next, we analyzed Osbpl2 expression in the organ of Corti of mice at the protein level using anti-Osbpl2 antibody in combination with either anti-prestin antibody used as an outer hair cell (OHC) marker or anti-otoferlin antibody used as an inner hair cell (IHC) marker. Osbpl2 was detected in stereocilia of both OHCs (Figure 4A,B) and IHCs (Figure 4C,D). We found no difference in expression of Osbpl2 between P20 and 6-month-old mice (not shown).

### Comparison of *Osbpl2* mRNA levels between wildtype and homozygous *Mir96* mutant mice

No significant difference was observed between wildtype and homozygote levels of *Osbpl2* mRNA in the organ of Corti (Additional file 1).

## Discussion

Mutations in approximately 30 genes have been implicated in ADNSHL (with variable evidence) [2]. The respective gene products fall into many different categories and comprise ion channels and transporters, motor molecules, components of the extracellular matrix, the cytoskeleton, adhesion complexes etc. [1].

Of the five heterozygous variants found in genes from the mapped candidate regions on chromosomes 12 and 20 in our family, the *OSBPL2* frameshift mutation represented a reasonable candidate for the ADNSHL-causing mutation (see results section). OSBPL2 does not belong to any of the above protein classes. It is part of a 12-member, evolutionarily highly conserved family of lipid binding/transfer proteins, the oxysterol binding proteins (OSBPs) and related proteins (OSBPLs) that share the characteristic OSBP signature, EQVSHHPP [38]. OSBPL proteins play an important role in non-vesicular intracellular transport of lipids, particularly oxysterol, a derivative of cholesterol. OSBPLs serve as sterol sensors and transporters that modulate the lipid composition of cell organelle membranes and assembly of protein complexes, thereby impacting signaling, vesicle transport and lipid metabolism [39].

*Osbpl2* was among 132 mRNAs with 3′UTRs predicted to contain potential target sites for *miR-96* [32], a microRNA whose mutations cause progressive hearing loss in mice and humans [32,40], but we found no upregulation of *Osbpl2* in *Mir96* mutant mice (*diminuendo*) (Additional file 1). However, besides its previously reported expression in the mouse organ of Corti at the onset of hearing [41], there are several lines of evidence that *OSBPL2* is the gene underlying ADNSHL in the family described herein: The frameshift mutation c.141_142delTG very likely represents a loss-of-function allele causing *OSBPL2* haploinsufficiency. It either results in a truncated non-functional protein of 151 residues (wild-type: 480 residues) including 102 unrelated amino acids (p.Arg50Alafs*103) or in an unstable mRNA undergoing nonsense-mediated decay. OSBPL2 interacts with diaphanous homologue 1 (DIAPH1) [42], the gene mutated in human ADNSHL type 1 (*DFNA1*) [43]. DIAPH1 is a Rho effector protein that regulates cytoskeletal dynamics by interacting with actin, microtubules and other proteins associated with cytoskeleton function [44,45]. Mutations in *DIAPH1* are thought to impair the structural integrity of hair cells’ stereocilia, which strongly depends on their actin cytoskeleton, and of the kinocilium, which is built around a microtubular backbone. As is assumed for DIAPH1, OSBPL2 could play a role for the maintenance of hair cells’ cytoskeleton, which would be compatible with the prominent presence of OSBPL2 protein at stereocilia (Figure 4). OSBPL2 binds phosphatidylinositol (3,4,5)-trisphosphate (PtdIns(3,4,5)*P*_3_) [46], a phospholipid of the plasma membrane that is crucial for defining neuronal polarity [47,48], a possible hint that OSBPL2 could be needed to establish and maintain polarity of hair cells. It remains to be determined if the interaction of both proteins is reflected by (at least partial) cellular co-localization.

Of note, an *OSBPL2* frameshift mutation in close proximity to the nucleotide position affected by the mutation reported herein has recently been described to co-segregate with ADNSHL in a large Chinese family [49]. Similar to the German *DFNA67* family, age of onset was variable (5 to 32 years), and hearing loss was progressive, ranging from mild to profound. This additional *DFNA67* family strongly supports the association of *OSBPL2* mutations with ADNSHL.

A truncating variant in a gene closely neighboring the chromosome 20 candidate locus, a nonsense mutation in *SLC17A9*, p.Ser96*, is unlikely to cause hearing loss because it was present in healthy individuals of both our family and our in-house database of 511 epilepsy exomes, and in the general population. *SLC17A9* encodes a vesicular nucleotide transporter [50], and heterozygous mutations in this gene have recently been reported to cause disseminated superficial actinic porokeratosis, DSAP [51]. None of the 12 individuals from our *DFNA67* family who carried the p.Ser96*_SLC17A9_ mutation had any skin abnormalities. In conclusion, *SLC17A9* is not only unrelated to hearing loss in this family; its haploinsufficiency does not seem to cause DSAP either. The manifestation of DSAP might thus only result from missense mutations with a dominant-negative effect. On the other hand, our data challenge the assumption that *SLC17A9* mutations cause DSAP, and additional research seems necessary to verify the postulated implication of *SLC17A9* in skin disease.

## Conclusions

The association of *OSBPL2* mutations with ADNSHL indicates a role of lipid metabolism in hair cell function, defining another functional category of proteins involved in hearing loss. However, further research is necessary to clarify how OSBPL2 deficiency causes hearing loss. Other members of the OSBPL family should be considered as potential candidates in future studies aimed at the identification of novel deafness genes.

## Additional file

Additional file 1:
**Comparison of** ***Osbpl2*** **mRNA levels between wildtype and homozygous** ***Mir96*** **mutant** ***diminuendo*** **(** ***Mir96*** ^***Dmdo***^ **) mice.** Quantitative real-time PCR on cDNA generated from normalised RNA from the organs of Corti of 4-day-old wildtype (blue) and *diminuendo* homozygote (red) littermates [32]. Error bars represent standard deviation. Quantities were normalised to *Hprt1* levels. No significant difference was observed (p = 0.083, Student’s *t*-test).
