# Supplementary figures and images for: OSBPL2 encodes a protein of inner and outer hair cell stereocilia and is mutated in autosomal dominant hearing loss (DFNA67)
Source: Orphanet J Rare Dis. 2015 Feb 10;10:15. doi: 10.1186/s13023-015-0238-5 (PMC4334766; doi:10.1186/s13023-015-0238-5)

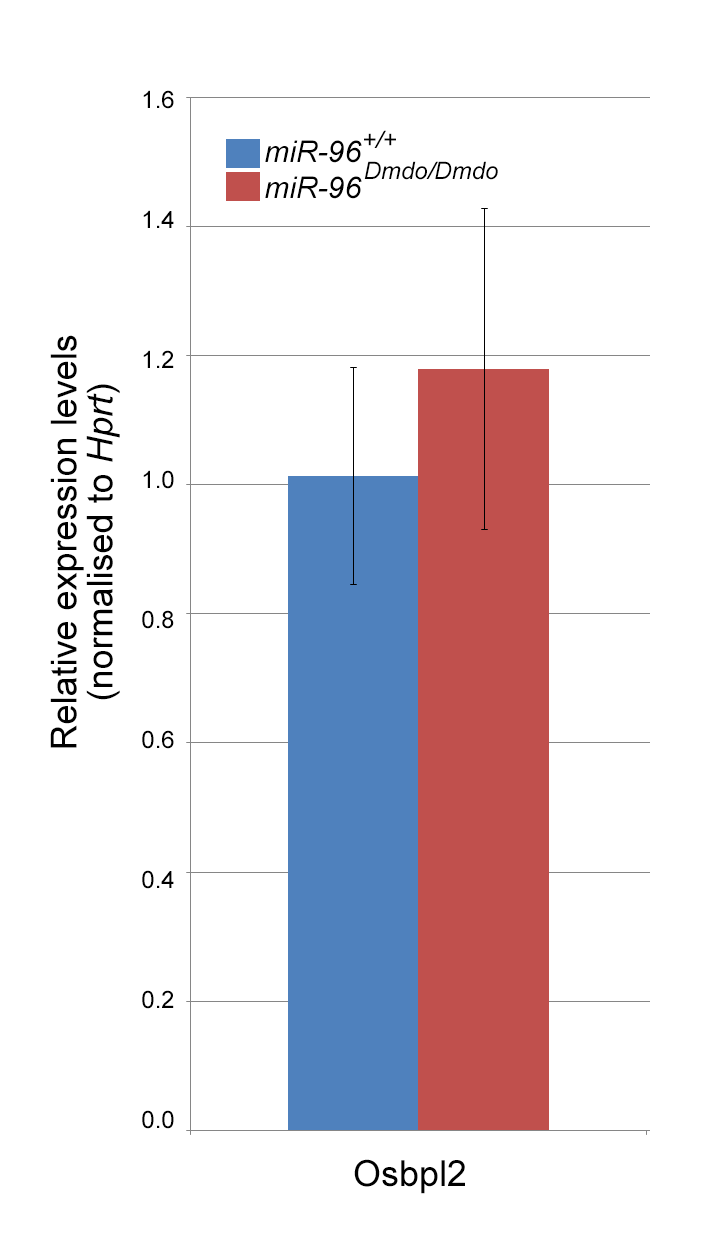

Supplement: Additional file 1: — Comparison of Osbpl2 mRNA levels between wildtype and homozygous Mir96 mutant diminuendo ( Mir96 Dmdo ) mice. Quantitative real-time PCR on cDNA generated from normalised RNA from the organs of Corti of 4-day-old wildtype (blue) and diminuendo homozygote (red) littermates [32]. Error bars represent standard deviation. Quantities were normalised to Hprt1 levels. No significant difference was observed (p = 0.083, Student’s t-test). [file 13023_2015_238_MOESM1_ESM.png]
